# Stigma toward people with COVID-19 among the Lebanese population: a cross-sectional study of correlates and mediating effects

**DOI:** 10.1186/s40359-021-00646-y

**Published:** 2021-10-22

**Authors:** Chadia Haddad, Sandrella Bou Malhab, Diana Malaeb, Hala Sacre, Danielle Saadeh, Vanessa Mourtada, Pascale Salameh

**Affiliations:** 1 Research Department, Psychiatric Hospital of the Cross, P.O. Box 60096, Jal Eddib, Lebanon; 2 INSERM, Univ. Limoges, IRD, U1094 Tropical Neuroepidemiology, Institute of Epidemiology and Tropical Neurology, GEIST, Limoges, France; 3 INSPECT-LB (Institut National de Santé Publique, d’Épidémiologie Clinique et de Toxicologie-Liban), Beirut, Lebanon; 4 School of Pharmacy, Lebanese International University, Beirut, Lebanon; 5 Faculty of Public Health, Lebanese University, Beirut, Lebanon; 6 Faculty of Letters and Human Sciences, Lebanese University, Fanar, Lebanon; 7 Faculty of Pharmacy, Lebanese University, Beirut, Lebanon; 8 University of Nicosia Medical School, Nicosia, Cyprus

**Keywords:** Stigma, Discrimination, Self-stigma, COVID-19, Pandemic

## Abstract

**Introduction:**

Stigma develops during outbreaks such as the COVID-19 pandemic due to the human fear that arises from the anxiety about a disease of an unknown etiology, with the associated detrimental consequences on both the individual and society. This study was conducted to assess if knowledge about COVID-19, attitude, practice and behavior regarding preventive measures against COVID-19, fear, and anxiety towards COVID-19 will affect the level of stigma and evaluate the mediating effect of fear, anxiety, and diagnosis of COVID-19 on stigma.

**Methods:**

A cross-sectional online survey conducted between December 20, 2020, and January 05, 2021, enrolled 405 participants recruited from the Lebanese population. Two scales were created and adapted to the Lebanese context to measure the current stigma (stigma discrimination scale, self-stigma scale) toward COVID-19.

**Results:**

More than half of the sample had moderate to severe stigma discrimination (62%) and self-stigma (65.9%). The multivariable analysis showed that higher fear of COVID-19 scale (Beta = .143) was significantly associated with a higher stigma discrimination scale. Whereas, higher knowledge score (Beta =  −.153) was significantly associated with a lower stigma discrimination scale. Fear of COVID-19, anxiety from COVID-19, being diagnosed with COVID-19, and having a family member with COVID-19 partially mediated the association between knowledge and stigma discrimination scale. No mediation effect of fear and anxiety scale was found between the knowledge and self-stigma score.

**Conclusion:**

Our main findings indicate that a considerable proportion of the Lebanese population has stigma discrimination behaviors toward COVID-19 patients and that those who were infected with the virus experienced COVID-19-related stigmatization.

**Supplementary Information:**

The online version contains supplementary material available at 10.1186/s40359-021-00646-y.

## Introduction

Today humanity is facing one of the biggest challenges of the century since the first case of coronavirus disease 2019 (COVID-19) was detected in China and classified as a pandemic. The novel coronavirus-2 (SARS-CoV2) is rapidly spreading and affecting millions of people worldwide, with a mortality rate of 2.2%, as reported by the World Health Organization (WHO) on February 2, 2021 [1]. In Lebanon, the first case of COVID-19 was diagnosed in February 2020; since then, the numbers have steadily increased, which mandated several lockdowns, in an attempt to limit virus transmission [2]. The nature widespread of COVID-19 has raised global concerns as, in the absence of effective treatment, therapy remains empirical and symptomatic [3–6]. Thus, to minimize the virus’ spread, efforts are focusing on prevention, including social distancing, awareness through public health education, and hygiene practices in daily routines, in addition to sanitary lockdown [7]. Moreover, this pandemic has had a psychological impact on people, manifested by anxiety, sadness, and depression [8].

Another notable factor associated with this pandemic is the stigma in its two dimensions (public stigma and self-stigma), previously documented with other infectious, physical, and psychological health disorders, especially when isolation and quarantine are involved [9]. Public stigma consists of prejudice, stereotypical beliefs, and discriminatory behaviors, such as disallowing COVID-19 patients from full community participation [10]. Self-stigma is the internalization of the negative views and feelings of others and social devaluation of the illness, occurring when individuals come to believe the negative societal conceptions and stereotypes associated with their condition [11]. Perceived devaluation and discrimination is thought to lead to diminished self-esteem and self-efficacy [12]. A person with an undesired condition is aware of public stigma about their condition, subsequently, the person concurs that these stereotypes apply to them and might lead to harm, to significant decreases in self-esteem and self-efficacy [12]. Believing that one belongs to a stigmatized group can lead to negative social comparisons, feelings of inferiority, inadequacy, and self-criticism [13]. Self-criticism could triggers the emotional response of thinking as being attacked, persecuted or feeling with anger, disgust or hate consequently increasing vulnerability, expression of symptoms and elevate risk of relapse of a disease [14]. Several studies have evaluated the association between personality traits and stigma [15–17]. For example a study done by Arikan among 700 university students have found a strong association between narcissistic personality and the tendency to stigmatize others [15]. Another study done by Brown among 605 college students found that openness and agreeableness were negatively associated with stigma towards severe mental illness [16].

Stigma or stigmatization develops during outbreaks due to the human fear that arises from the anxiety about a disease of an unknown etiology, with the associated detrimental consequences on both individual and society levels [18]. In many countries, the dramatic global increase in the number of persons infected with the COVID-19 virus has raised public fear and concerns [19]. Thus, a new form of discrimination emerged in some societies against individuals infected or in close contact with patients with COVID-19 [19]. Also, anxiety and worry for being stereotyped has shown to lead to delaying or even masking the diagnosis by postponing the sought of healthcare services of symptomatic patients and under-detecting infectious people [20]. Delayed diagnosis has been associated with prognostic deterioration due to an increase in the viral load, mainly in the elderly and vulnerable groups facilitating the rapid spread of both COVID-19 and its complications [21].

The level of stigma associated with COVID-19 is based on many factors, as it is a recent disease surrounded by many controversies, and people are often afraid of the unknown, associating their fear with other infected people [22]. Some persons became fearful of suspicious or infected persons as they are actual risk factors for COVID-19 disease and they held negative attitudes and beliefs toward them [23]. Stigma or discriminatory behaviors may increase due to a lack of knowledge of the novel coronavirus disease, means of transmission, effective treatment options, and preventive strategies [18, 24]. Stigma towards COVID-19 is due to the fear of its mortality and high communicability that can be resolved through proper education and transparent healthcare policies [25]. Also, excessive misinformation could act as a driver or facilitator of stigmatization linked to COVID-19 [26]. Thus, improving knowledge would reduce stigma perceptions, particularly among vulnerable groups, including low-income, low-educated, rural residents, and older people [25]. Previous literature has also highlighted that people with better personal resources, such as higher income, higher educational level, better social support, and good mental health, are more knowledgeable about emerging infectious diseases, thus less likely to stigmatize [27, 28]. Furthermore, stigma has been practiced more in some communities, where people are blamed and criticized for spreading the virus [29].

Several countries have reported stigma associated with COVID-19, which may dramatically increase the level of stress when information is disclosed on social media platforms [30, 31]. However, there is scarce COVID-19-specific stigma research despite the fact that stigma can affect health outcomes. Most of the scientific articles found in the literature are in the form of a review, letter to the editor or commentary [30–33]. Only two studies to our knowledge have been found in the Arab countries that assessed the stigma level toward COVID-19 patients [34, 35]. A study done in Jordan among 1655 participants from the general population have found that the prevalence of stigma towards infected people and their contact was 64% [35]. Another study done in Egypt among 509 physicians have found that 31.2% of participants reported severe level of COVID-19-related stigma [34]. Thus, it is crucial to evaluate factors associated with stigma since it can undermine family connections, weaken society cohesion, and prompt social isolation of groups, resulting in more severe health problems and difficulties controlling a disease outbreak [36].

**Fig. 1:**
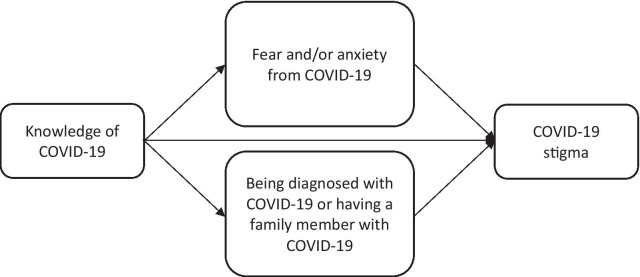
A model assessing the relationships between knowledge, fear and/or anxiety of COVID-19, being diagnosed with COVID-19 or having a family member with COVID-19, and stigma

Outbreaks of infectious diseases such as the COVID-19 are often related to a greater fear of contracting the disease, which can lead to emotions of anxiety and mistrust among the general people [37]. As a result of this fear and anxiety, some communities have begun to discriminate against people who are infected with COVID-19 or who get the disease [38]. Consequently, stigma arose from a lack of understanding or fear and anxiety about a disease, which is defined as negative attitudes and beliefs about people, places, or things [23]. In addition, those who contracted the virus might feel judged by others or by themselves and might hide their illness to avoid discrimination [30]. The COVID-19 patients were accused of being ignorant and careless, and hence are considered responsible for contracting the virus [30]. The COVID-19 patients were stereotyped as coronavirus spreaders who were actively spreading the virus [30]. As a result of this misconception, society adopted a number of negative behaviors and discriminative attitudes against them [30]. Therefore, stigma could be a consequence of a lack of understanding of how COVID-19 spreads, a desire to blame someone, fear of disease and concerns from the unknown, and gossip that spreads rumors and myths [23]. Based on this information and since no previous theoretical framework was found, a conceptual model was specially constructed for this study to understand the directional association between knowledge, fear, anxiety, being diagnosed with COVID-19, having a family member with COVID-19 and stigma (Fig. 1).

In Lebanon, no previous research has assessed stigma toward COVID-19 and its associated factors. Therefore, this study aims to explore whether knowledge about COVID-19, attitude, practice and behavior regarding preventive measures against COVID-19, fear, and anxiety towards COVID-19 will affect stigma levels and evaluate the mediating effect of fear, anxiety, and diagnosis of COVID-19 on stigma.

## Methods

### Study design and sampling

A cross-sectional online survey conducted between December 20, 2020, and January 05, 2021, enrolled 405 participants recruited from the Lebanese population. In this study, we report all measures, manipulations and exclusions. Data collection was carried out through an anonymous self-administered questionnaire developed on Google Forms and shared on various social media platforms (WhatsApp, Facebook, and Instagram)

(https://forms.gle/jQXG1E3ScHbhDb5W6) (Additional File 1), using the snowball sampling technique. In turn, participants were encouraged to share the form with their friends and contacts. All people above 18 years with access to the internet were eligible to participate. All participants were aware of the general purpose of the study and gave prior informed consent. Participation in this study was voluntary, and participants received no incentive in return.

### Sample size calculation

The Epi Info™ software (Centers for Disease Control and Prevention, Epi Info™) calculated a minimum sample of 354 participants, considering a Lebanese population of 6,856,000 [39] and a prevalence of 64% of individuals having stigma towards people with COVID-19 (based on a Jordan study [35]), with a 95% confidence level, and an alpha error of 5%. The target was 390 participants after adding 10% (*n* = 35.4) to take into account non-response or missing data; the final sample included 405 participants. In the absence of similar research in Lebanon, all the calculations were based on the Jordanian study. Sample size was determined before any data analysis.

### Questionnaire

The online survey tool, available in English and Arabic, included open and closed-ended questions and consisted of two parts.

The first part of the questionnaire consisted of sociodemographic characteristics such as age, gender, marital status, educational level, monthly income, employment status, the region of residence, number of people living in the house, number of rooms in the house, and religion. It also included questions related to direct or indirect contact with infected people, in addition to diagnosis and testing status with COVID-19. The household crowding index was calculated by dividing the number of persons living in the house by the number of rooms, excluding the kitchen and bathrooms. The monthly income was divided into four levels: no income, low < 1000 USD (United States Dollar), intermediate 1000–2000 USD, and high income > 2000 USD. The official exchange rate was considered: 1 USD = 1500 Lebanese Pounds (LBP). To differentiate between those who have been diagnosed with COVID-19, a dichotomized question was asked as follows: “Have you been diagnosed with COVID-19? (Yes or No)”. Also, a question was asked if people have ever gotten tested for COVID-19 as follows: “Have you been tested for COVID-19? (Yes or No)”.

The second part of the questionnaire consisted of several scales used in this study:

#### Stigma scales

Two scales were developed and used according to the type of stigma: the stigma discrimination scale and the self-stigma scale.

At the time of the study, no particular tools for assessing the COVID-19-related stigma were available in the literature, except for one instrument related to COVID-19 self-stigma among healthcare workers after quarantine in Vietnam [40]. Therefore, other instruments used to measure self-stigma and stigma discrimination in the general population in other viral epidemics were sought, and the only available were those used during the HIV/AIDS pandemic [41–44]. In 2004, Verma et al. [45] created a scale, based on the modified HIV Berger scale [44], to measure stigma among healthcare workers during the SARS pandemic. Thus, after a thorough literature review, we created and adapted two scales to measure stigma discrimination and self-stigma during COVID-19.

#### The Stigma Discrimination Scale (SDS-11)

It consists of 11 items selected from previous studies [41–43] and measures the discriminatory attitude toward people with COVID-19. All items are rated on a 5-point Likert scale from 1 (*Strongly disagree*) to 5 (*Strongly agree*). Examples of the items include: “You feel it is not worthwhile for you to serve persons who contracted COVID-19,” “People with current COVID-19 are dangerous to the society,” and “People with current COVID-19 should not have the same freedoms as other people.” The total stigma discrimination score calculated by summing all the answers ranges from 11 to 55, with higher scores indicating a higher level of stigma discrimination. In this study, the scale showed excellent reliability with a Cronbach’s alpha of 0.917.

#### The Self-Stigma Scale (SSS-15)

This tool includes 15 items selected from COVID-19-related stigma [40] and the HIV Berger scale [44]; it measures self-stigma (i.e., the negative attitudes, including internalized shame about own condition) in people previously or currently infected with COVID-19 only. The items are rated on a 5-point Likert scale from 1 (*Strongly disagree*) to 5 (*Strongly agree*). Examples of the items include: “I feel guilty because of being isolated,” “People have physically backed away from me when they learned I have COVID-19,”and “I have been hurt by how people reacted to learning I have COVID-19”. The total self-stigma score, ranging from 15 to 75, is the sum of answers to all 15 items. Higher scores indicate higher levels of self-stigma. In this study, the scale showed excellent reliability with a Cronbach’s alpha of .917.

In the absence of a cut-off value for any stigma scale worldwide, the percentile was used to categorize both scales. This approach suggested by Charles et al. [46] in their study of stigma toward HIV/AIDS, categorized people into three groups: no or mild, moderate, and severe stigma, using the 33rd and 66th percentile cut-off values from the distribution of scores.

Thus, in our study, both stigma scales were classified into three categories: 0 to 33% (no or mild stigma), 33 to 66% (moderate stigma), and > 66% (severe stigma).

#### Fear of COVID-19 scale (FCV-19S)

The FCV-19S is a 7-item scale developed to assess the fear of COVID-19 among the general population [47]. In this study, the Arabic validated version of the FCV-19S was used [48]. Examples of the items include: “I am most afraid of coronavirus-19,” “My hands become clammy when I think about coronavirus-19,” “I cannot sleep because I’m worrying about getting coronavirus-19.” Items are rated on a 5-point Likert scale from 1 (*Strongly disagree*) to 5 (*Strongly agree*). A total score is calculated by adding up each item score with a higher score indicating greater fear of COVID-19 [47]. In this study, the scale showed acceptable reliability with a Cronbach’s alpha of 0.856.

#### Coronavirus Anxiety Scale (CAS)

The CAS is a self-report 5-item scale, measuring physiologically-based symptoms triggered by information and thoughts relevant to COVID-19 [49]. Participants are asked to rate how often they experienced each symptom of anxiety over the past two week. The measure is scored on a 5-point Likert scale from 0 (*Not at all*) to 4 (*Nearly every day*). Examples of the items include: “I felt dizzy, lightheaded, or faint, when I read or listened to news about the coronavirus,” “I felt paralyzed or frozen when I thought about or was exposed to information about the coronavirus,” and “I felt nauseous or had stomach problems when I thought about or was exposed to information about the coronavirus.” The total score is calculated by summing the five items, with higher scores indicating higher anxiety toward COVID-19 [49]. In this study, the scale showed good reliability with a Cronbach’s alpha of 0.846.

#### Knowledge, attitude, and practice (KAP) toward the COVID-19 pandemic

The questions used to assess KAP toward COVID-19 were selected from previous studies conducted among health professionals and the general population [50–56] and adapted to this research.

*Knowledge sub-scale:* Twenty multiple-choice items (with single- and multiple-option answers) were used in this section to assess the knowledge regarding the COVID-19 disease. All answers were coded as binary variables (1 = Yes, 0 = No); multiple-option questions were considered as separate variables. Examples of the items include: “For how long should a person be isolated in case of COVID-19 infection suspicion (mild symptoms or contact with an infected persons),” “If a suspected person tests negative but has no symptoms,” and “Can someone who has been quarantined for COVID-19 spread the illness to others?”. The total was calculated by summing all the correct answers ranged from 0 to 29, where a higher score would indicate higher knowledge about COVID-19. In this study, the scale showed acceptable reliability with a Cronbach’s alpha of 0.557.

*Attitude sub-scale:* Six questions assessed the positive attitudes toward preventive measures, adherence to government actions, and adaptive steps toward COVID-19. All questions are measured on a 3-point Likert scale from 1 (*Disagree*) to 3 (*Agree*). Examples of the items include: “Do you think social distancing/self-isolation is an effective measure to reduce the spread of COVID-19?” “Keeping up with the information regarding the government’s call for COVID-19 preventive efforts is important for the community,” and “People with COVID-19 who isolate themselves show that they have a responsibility in preventing the transmission of COVID-19”. The total attitude score created by summing the six answers ranged from 6 to 18, where a higher score would indicate a more positive attitude towards COVID-19. In this study, the scale showed acceptable reliability with a Cronbach’s alpha of 0.615.

*Practice sub-scale:* Twenty-four items evaluated positive practice and behavior regarding preventive measures against COVID-19. All questions were rated on a 5-point Likert scale from 1 (*Never*) to 5 (*Always*). Examples of the items include: “In the last few days, have you worn a mask when you were in a crowded place?” “Do you maintain social distancing (or home quarantine)?” and “Do you clean/disinfect your mobile phone?” The total practice score calculated by summing the 24 items ranged from 24 to 120, with higher scores indicating appropriate safety practice towards COVID-19. In this study, the scale showed excellent reliability with a Cronbach’s alpha of 0.900.

### Translation procedure

All the scales, except the FCV-19S scale, were translated from English into Arabic, following the forward and backward translation method. Two authors performed the translation from English into Arabic, and another two did the back-translation. Discrepancies were resolved by consensus between the original English edition and the translated one.

A pilot study was conducted on ten subjects to check the clarity of the questionnaire and test for the acceptability of questions. Related data were included in the final dataset and they did not affect neither negatively or positively on the current study result.

### Statistical analysis

Data were analyzed on SPSS software version 25. Cronbach’s alpha was calculated for the reliability analysis of all scales. A descriptive analysis was performed using absolute frequencies and percentages for categorical variables and means and standard deviations (SD) for quantitative measures. Student’s *t*-test or ANOVA *F* tests were used for categorical variables with two or more levels, respectively, to assess the association of variables with continuous stigma scales. The Pearson correlation coefficient “r” was used to measure the association between continuous variables.

Construct validity of the two stigma scales was assessed using a principal component analysis. To ensure the model’s adequacy, Kaiser–Meyer–Olkin measure of sampling adequacy and Bartlett’s test of sphericity were calculated. Factors with eigenvalues values larger than one were retained and the scree plot method was used for determining the number of components to extract [57]. Only items with factor loading larger than 0.4 were considered [58]. Moreover, the internal consistency of the stigma scales was assessed using Cronbach's alpha.

Three linear regressions were performed, taking the stigma discrimination scale and the self-stigma scale as the dependent variables. The stepwise method was used to simultaneously remove the weakest correlated variables and come up with a model that best explains the distribution. The unstandardized coefficient “Beta” was used to measure the effect of the factors on the dependent variables. All the variables that showed a *p*-value < 0.2 in the bivariate analysis were included in the model to eliminate potential confounding factors.

The Johnson-Neyman technique (CAHOST method) was used to perform a moderation analysis where explanatory variables interact to affect a desired response [59]. Four models were used to assess the effect of knowledge on stigma discrimination. This effect depending on fear from COVID-19 (model 1), anxiety from COVID-19 (model 2), being diagnosed with COVID-19 (model 3) and the diagnosis of a family member with COVID-19 (model 4). The CAHOST method generated simple slopes graphs and 95% confidence intervals (CI) to test the significance of the effect [59]. The moderating effect was significant when the CI did not include zero [59]. The covariates included in the models showed significant associations with the stigma discrimination scale in the bivariate analysis.

The PROCESS SPSS Macro version 3.4 model four was used to calculate three pathways in the mediation analysis. Pathway A determined the regression coefficient for the effect of knowledge on fear, anxiety, and diagnosis of COVID-19. Pathway B examined the association between anxiety, fear, and diagnosis of COVID-19 on stigma, independent of the knowledge level, and pathway C estimated the total and direct effect of knowledge on stigma. Pathway AB calculated the indirect intervention effects. The macro generated bias-corrected bootstrapped 95% confidence intervals (CI) to test the significance of the indirect effect. Mediation was significant when the CI around the indirect effect did not include zero. The covariates that were included in the mediation model were those that showed significant associations with the stigma scales in the bivariate analysis. A *p*-value less than 0.05 was considered significant.

## Results

Table 1 summarizes the sociodemographic characteristics of the participants. The mean age of the participants was 28.38 (SD = 12.02) years, and the mean household crowding index was 1.14 (SD = 0.55). The majority of the participants were females (79.8%), single (68.1%), had a university education level (89.6%), lived in urban areas (73.3%), and had low and no income (58.8%). More than half of the participants were unemployed (55.8%), and only 28.1% had a family member working in the medical field. Only 10.1% of the participants were diagnosed with COVID-19, and only 27.9% had a history of COVID-19 in the family. More than half of the sample had moderate to severe stigma discrimination (62%) and self-stigma (65.9%).

**Table 1:** Sociodemographic characteristics of the study sample (N = 405)

	Frequency	Percentage
Gender		
Male	82	20.2%
Female	323	79.8%
Marital status		
Single	276	68.1%
Married	129	31.9%
Education level		
School level	42	10.4%
University level	363	89.6%
Monthly income		
No income	185	45.7%
Low	53	13.1%
Intermediate	75	18.5%
High	92	22.7%
Employment status		
Employed, medical field (frontline contact with COVID-19 patients)	10	2.5%
Employed, medical field (non-frontline contact)	55	13.6%
Employed, non-medical	114	28.1%
Unemployed	226	55.8%
Family member in the medical field		
Yes	114	28.1%
No	291	71.9%
Living place		
Rural	108	26.7%
Urban	297	73.3%
Religion		
Christian	71	17.5%
Muslim	254	62.7%
Druze	42	10.4%
Atheist	1	0.2%
Refuse to answer	35	8.6%
Other	2	0.5%
Diagnosed with COVID-19		
Yes	41	10.1%
No	364	89.9%
Tested with COVID-19		
Yes	164	40.5%
No	241	59.5%
History of COVID-19 in the family		
Yes	113	27.9%
No	277	68.4%
I do not know	15	3.7%
Time spent on COVID-19 information sources/day		
0–30 min	308	76.0%
> 30 min	97	24.0%
	Mean	SD
Age	28.38	12.02
Household crowding index	1.14	.55

### Description of the scales used in the study

Table 2 describes all the scales used in this study in terms of mean, standard deviation (SD), median, minimum, and maximum.

**Table 2:** Description of the scales used

	Mean	SD	Median	Minimum	Maximum
Stigma discrimination scale	26.25	5.41	26.00	12.00	41.00
Self-stigma scale	38.65	12.46	39.00	15.00	74.00
Fear of COVID-19 scale	17.5	5.5	17.0	7.0	32.0
COVID-19 Anxiety scale	1.2	2.4	1.0	.0	16.0
Knowledge of COVID-19 scale	20.4	3.5	21.0	5.0	27.0
Positive attitude toward COVID-19 scale	16.5	1.8	17.0	6.0	18.0
Positive practice of COVID-19 scale	104.6	12.5	109.0	50.0	120.0

### Factor analysis

A factor analysis using a principal component analysis technique was used to test the validity of the COVID-19 stigma discrimination scale and self-stigma scale and ensure the model’s adequacy. All items of the two scales could be extracted from the list and none of them were removed because no item over-correlated to each other (*r* > 0.9), had a low loading on factors (< 0.3) or a low communality (< 0.3). The Kaiser–Meyer–Olkin measure of sampling adequacy was 0.641 for the stigma discrimination scale and 0.717 for the self-stigma scale and Bartlett’s test of sphericity was significant (*p* < 0.001) for the two scales. Moreover, the COVID-19 stigma discrimination scale items produced four factors that had an eigenvalue over 1, the first factor explained 22.88% of the total variance, while the second explained 15.58%, the third 12.46%, and the fourth 10.20% making a total of 61.13% of the data variance. The total reliability of Cronbach’s alpha was 0.565 (Additional file 1: Table S1). The COVID-19 self-stigma scale items produced three factors that had an eigenvalue over 1, the first factor explained 46.69% of the total variance, while the second explained 16.54%, and the third 10.25% making a total of 56.16% of the data variance. The total reliability of Cronbach’s alpha was 0.917 (Additional file 1: Table S2).

### Bivariate analysis

The bivariate analysis taking the stigma discrimination as the dependent variable showed that lower scores were associated with being diagnosed with COVID-19 (*M* = 23.59; *SD* = 5.82) compared to not (*M* = 26.55; *SD* = 5.30), having tested for COVID-19 (*M* = 25.03; *SD* = 5.22) compared to not (*M* = 27.09; *SD* = 5.40), having had direct contact with COVID-19 patient (*M* = 25.39; SD = 5.44) compared to not (*M* = 26.44; *SD* = 5.36), having had direct contact with suspected COVID-19 case (*M* = 25.22; *SD* = 4.80) compared to not (*M* = 26.67; *SD* = 5.69) and having a family history of COVID-19 (*M* = 23.95; *SD* = 5.08) compared to not (*M* = 27.12; *SD* = 5.33). Whereas, higher stigma discrimination scores were significantly associated with being a health care worker (*M* = 25.88; *SD* = 5.44) compared to not (*M* = 28.22; *SD* = 4.88), fear of COVID-19 (*r* = 0.127, *p* = 0.010), and higher anxiety of COVID-19 (*r* = 0.118, *p* = 0.017). However, a higher knowledge score (*r* = -0.109, *p* = 0.028) was significantly correlated with a lower stigma discrimination score (Table 3).

**Table 3:** Bivariate analysis taking the COVID-19 stigma discrimination and self-stigma as the dependent variables

	COVID-19 stigma discrimination		COVID-19 self-stigma	
	Mean (SD)	*p* value	Mean (SD)	*p* value
Ever diagnosed with COVID-19				
No	26.55 (5.30)	.001**		
Yes	23.59 (5.82)			
Effect Size Cohen’s d	− .532			
Ever tested for COVID-19				
No	27.09 (5.40)	< .001***		
Yes	25.03 (5.22)			
Effect size Cohen’s *d*	− .388			
Gender				
Male	26.96 (5.11)	.185	32.38 (12.48)	.113
Female	26.07 (5.49)		40.18 (12.16)	
Effect size Cohen’s *d*	− .168		.633	
Have a family member working in the medical field				
No	25.97 (5.34)	.091	37.50 (12.55)	.389
Yes	26.98 (5.58)		41.15 (12.40)	
Effect size Cohen’s *d*	.185		.293	
Ever being quarantined				
No	26.70 (5.52)	.072	35.17 (18.07)	.612
Yes	25.72 (5.26)		39.25 (11.50)	
Effect size Cohen’s *d*	− .182		.269	
Marital status				
Single	25.88 (5.68)	.032*	39.90 (11.82)	.298
Married	27.05 (4.73)		35.27 (14.11)	
Effect size Cohen’s *d*	.224		− .356	
Income level				
No income	26.29 (5.50)	.989	41.78 (14.15)	.091
Low income	26.38 (5.11)		40.43 (8.75)	
Intermediate income	26.07 (6.20)		37.58 (9.19)	
High income	26.27 (4.80)		24.75 (12.12)	
Effect size Cohen’s *d*	.118		.138	
Education level			–	
Primary	39.00 (00)	< .001***	–	.277
Complementary	24.20 (6.34)		–	
Secondary	26.62 (4.70)		44.40 (12.42)	
University	26.14 (5.37)		37.86 (12.42)	
Effect size Cohen’s *d*	.143		.145	
Employment status				
Medical field	28.22 (4.88)	.001**	26.80 (7.76)	.012*
Non-medical field	25.88 (5.44)		40.31(12.16)	
Effect size Cohen’s *d*	− .453		1.324	
Religion				
Christian	26.49 (4.41)	.004**	31.00 (8.49)	.02*
Muslim	26.28 (5.61)		43.04 (11.91)	
Druze	28.24 (5.96)		23.00 (13.86)	
Refuse to answer	23.66 (4.12)		36.33 (9.24)	
Other	19.50 (3.54)		35.50 (.70)	
Effect size Cohen’s *d*	.244		.287	
Had an indirect contact with COVID-19 a patient				
No	26.94 (5.55)	.062	27.33 (15.29)	.043*
Do not know	25.93 (5.58)		38.50 (7.84)	
Yes	25.60 (5.16)		41.22 (11.88)	
Effect size Cohen’s *d*	.189		.132	
Had a direct contact with a COVID-19 patient				
No	26.44 (5.36)	.017*	32.75 (13.86)	.129
Do not know	28.27 (5.26)		43.50 (8.29)	
Yes	25.39 (5.44)		40.48 (11.92)	
Effect size Cohen’s *d*	.147		.109	
Had a direct contact with a suspected COVID-19 individual				
No	26.67 (5.69)	.032*	33.95 (12.68)	.057
Do not know	27.17 (5.07)		42.00 (4.24)	
Yes	25.22 (4.80)		43.26 (11.25)	
History of COVID-19 in the family				
No	27.12 (5.33)	< .001***	37.22 (15.71)	.589
I don’t know	27.60 (4.58)		–	
Yes	23.95 (5.08)		38.68 (11.65)	
	.132		.130	
Time spent on COVID-19 information sources/day				
0–30 min	25.99 (5.15)	.089	37.69 (10.79)	.544
> 30 min	27.08 (6.25)		44.33 (20.16))	
Effect size Cohen’s *d*	.19		.411	
	*r*	*p* value	*r*	*p* value
Fear of COVID-19 scale	.127	.010*	.193	.227
COVID-19 Anxiety scale	.118	.017*	.115	.475
Practice total scale	− .069	.163	− .096	.551
Attitude total scale	− .014	.772	.106	.509
Knowledge score	− .109	.028*	− .249	.117

Statistical tests used: Student’s *t*-test or ANOVA *F* tests were used for the association between categorical variables with two or more levels and the stigma continuous scale. The Pearson correlation coefficient was used to measure the association between continuous variables

*SD* standard deviation, *r* correlation coefficient

* < .05; ** < .01; *** < .001

Regarding the self-stigma scale, lower scores were associated with being a healthcare worker (*M* = 26.80; *SD* = 7.76) compared to not (*M* = 40.31; *SD* = 12.16), while having had indirect contact with COVID-19 case (*M* = 41.22; *SD* = 11.88) compared to not (*M* = 27.33; *SD* = 15.29) scored higher on the self-stigma scale (Table 3).

### Multivariable analysis

A first linear regression taking the stigma discrimination scale as the dependent variable showed that higher fear of COVID-19 scale (Beta = 0.143), being a Druze (Beta = 0.157), and being married (Beta = 0.123) were significantly and positively associated with higher SDS scores. Whereas, higher knowledge score (Beta =  −0.153) was significantly associated with a lower SDS score (Table 4, Model 1).

**Table 4:** Multivariable analysis

Model 1: Linear regression taking the stigma discrimination scale in the whole sample as the dependent variable				
Factor	Standardized beta	Unstandardized beta	95% CI LL; UL	*p*-value
Fear of COVID-19	.143	.140	.047;.234	.003**
Religion Druze versus Christian	.157	2.793	1.089; 4.498	.001**
Knowledge score	− .153	− .236	− .386; − .086	.002**
Marital status (married vs. single*)	.123	1.430	.306; 2.553	.013*
Variables entered: marital status, education level, religion, fear scale, anxiety scale and knowledge scale				

Model 2: Linear regression taking the stigma discrimination scale in the whole sample as the dependent variable and adding the diagnosis of COVID-19 and having a family member with COVID-19 as the independent variables				
	Standardized beta	Unstandardized beta	95% CI LL; UL	*p*-value
History of COVID-19 in the family versus no	− .284	− 2.881	− 4.289; − 1.472	< .001***
Employed in medical field versus non-medical field	− .410	− 4.091	− 5.392; − 2.790	< .001***
Knowledge score	− .209	− .310	− .490; − .131	.001**
Direct contact with suspected COVID-19 case versus no	− .208	− 2.000	− 3.267; − .734	.002**
Religion Druze versus Christian	.267	2.572	1.238; 3.906	< .001***
Religion Muslim versus Christian	.167	2.673	.654; 4.692	.010*
Diagnosed with COVID-19	− .144	− 2.192	− 4.078; − .306	.023*
Marital status (married vs. single*)	.144	1.385	.143; 2.628	.029*
Variables entered: diagnosed for COVID-19, tested for COVID-19, gender, family member working in the medical field, being quarantined, marital status, education level, employment status, religion, indirect contact with COVID-19 patient, direct contact with COVID-19 patient, direct contact with suspected case, history of COVID-19 in the family, time spent on COVID-19 information, fear scale, anxiety scale, practice scale, knowledge scale				

Model 3: Linear regression taking the self-stigma scale in participants who were diagnosed with COVID-19 as the dependent variable				
Factor	Standardized beta	Unstandardized beta	95% CI LL; UL	*p*-value
Religion Muslim versus Christian	12.249	.479	5.702; 18.796	.001**
Indirect contact with COVID-19 case versus no contact	8.162	.314	1.500; 14.825	.018*
High income versus no income	− 12.157	− .293	− 22.773; − 1.542	.026*

Variables entered: Gender, income, employment status religion, indirect contact with COVID-19 patient, direct contact with COVID-19 patient, direct contact with suspected case, knowledge score

*LL* lower level, *UL* upper level

* < .05; ** < .01; *** < .001

A second linear regression taking the stigma discrimination scale as the dependent variable and adding the diagnosis of COVID-19 and having a family member with COVID-19 as independent variables showed that higher knowledge (Beta =  −0.209), having a history of COVID-19 in the family (Beta =  −0.284), being employed in the medical field (Beta =  −0.410), having direct contact with suspected COVID-19 case (Beta =  −0.208), and being diagnosed with COVID-19 (Beta =  −0.144) were significantly associated with lower SDS scores. However, being a Muslim (Beta = 0.167), a Druze (Beta = 0.267), and married (Beta = 0.144) were significantly associated with higher SDS scores (Table 4, Model 2).

A second linear regression taking the self-stigma scale as the dependent variable in patients diagnosed with COVID-19 showed that being a Muslim (Beta = 12.294) and having indirect contact with COVID-19 case (Beta = 8.162) were significantly and positively associated with self-stigma; whereas, a high income (Beta =  −12.157) was significantly associated with lower self-stigma (Table 4, Model 2).

### Moderation Johnson–Neyman analysis

The moderation analysis showed the following (results presented in Addtional file 1, supplementary file 3, Table S1 and Table S2):

Model 1 shows the effect of knowledge on stigma discrimination. The effect of knowledge depended on fear from COVID-19 in a way that in case of low fear scores, a high knowledge score was associated with a lower stigma discrimination (Additional file 1: Figs. S1 and S2).

Model 2 shows the effect of knowledge on stigma discrimination. The effect of knowledge depended on anxiety from COVID-19 in a way that in case of high anxiety scores, a high knowledge score was associated with a lower stigma discrimination (Additional file 1: Figs. S3 and S4).

Model 3 shows the effect of knowledge on stigma discrimination. The effect of knowledge depended on COVID-19 diagnosis result, in a way that in the case of positive COVID-19 diagnosis, a high knowledge score was associated with a lower stigma discrimination. (Additional file 1: Fig. S5 and S6).

Model 4 shows the effect of knowledge on stigma discrimination. The effect of knowledge depended on the diagnosis result of a family member with COVID-19 in a way that in the case of positive diagnosis, a high knowledge score would have been associated with a lower stigma discrimination but the moderation effect was not significant (Additional file 1: Figs. S7 and S8).

### Mediation analysis

Table 5 shows the mediating effect of fear and anxiety of COVID-19 between knowledge and stigma discrimination scale.

**Table 5:** Mediation analysis

Step 1: Taking the stigma discrimination scale as the dependent variable and the fear from COVID-19 scale as a mediation factor										
	Mediating variable			Dependent variables						Mediating effect of Fear from COVID-19
Predictors	Fear from COVID-19 scale			Stigma discrimination scale						
	Beta [LL, UL 95%CI]	t	p	Beta [LL, UL 95%CI]	t	p	Beta [LL, UL 95%CI]	t	p	
Model 1: taking the Knowledge score as the independent variable										
Knowledge score	.197 [− .059; .455]	1.51	.131	− .351 [− .561; − .141]	− 3.308	.001	− .35 [− .56; − .15]	− 3.39	.001	51.86
Fear from COVID-19				.130 [.035; .224]	2.694	.007				

Step 2: Taking the stigma discrimination scale as the dependent variable and the anxiety from COVID-19 scale as a mediation factor										
Predictors	Mediating variable			Dependent variables						Mediating effect of Anxiety from COVID-19
	COVID-19 anxiety scale			Stigma discrimination scale						
	Beta [LL, UL 95%CI]	t	p	Beta [LL, UL 95%CI]	t	p	Beta [LL, UL 95%CI]	t	p	
Model 2: taking the Knowledge score as the independent variable										
Knowledge score	− .115 [− .182; − .048]	− 3.359	.0009	− .142 [− .294: .010]	− 1.838	.067	− .35 [− .56; − .15]	− 3.39	.001	15.64
Anxiety from COVID-19				.229 [.011; .447]	2.066	.039				

Step 3: Taking the stigma discrimination scale as the dependent variable and being diagnosed with COVID-19 as a mediation factor										
	Mediating variable			Dependent variables						Mediating effect of being diagnosed as COVID-19
Predictors	Being diagnosed as COVID-19			Stigma discrimination scale						
	Beta [LL, UL 95%CI]	t	p	Beta [LL, UL 95%CI]	t	p	Beta [LL, UL 95%CI]	t	p	
Knowledge score	.01 [− .004; − .02]	1.47	.142	− .31 [− .51; − .11]	− 3.08	.002	− .35 [− .56; − .15]	− 3.39	.001	34.01
Being diagnosed as COVID-19				− 3.59 [− 5.56; − 1.62]	− 3.60	< .001				

	Mediating variable			Dependent variables						Mediating effect of history of COVID-19 among the family member
Predictors	History of COVID-19 among the family member			Stigma discrimination scale						
	Beta [LL, UL 95%CI]	t	p	Beta [LL, UL 95%CI]	t	p	Beta [LL, UL 95%CI]	t	p	
Knowledge score	.01 [− .009; − .03]	1.19	.23	− .30 [− .48; − .11]	− 3.17	.002	− .35 [− .56; − .15]	− 3.39	.001	98.86
History of COVID-19 among the family member				− 4.14 [− 5.39; − 2.88]	− 6.51	< .001				

Beta: unstandardized beta coefficient, t: is the ratio of the difference between the sample mean and the given number to the standard error of the mean

*p* p-value, *LL* lower level, *UL* upper level

Fear of COVID-19, anxiety from COVID-19, being diagnosed with COVID-19, and having a family member with COVID-19 partially mediated the association between knowledge and stigma discrimination scale.

## Discussion

Diseases such as COVID-19 influence both the medical condition and mental health. People may experience discrimination, stigma, fear, guilt, and shame, affecting their mental condition and causing severe psychological issues. Our study evaluated the level of stigma among a sample of the Lebanese population and assessed factors related to stigma. Our findings revealed that 62% of the people discriminate against COVID-19 patients and that 65.9% of the participants who were infected with COVID-19 experienced self-stigma, similar to those of a study about COVID-19-related stigmatization among a sample of Egyptian healthcare workers, reporting 61% of stigma towards healthcare workers and 57.5% of self-stigma [34]. Another study done in Jordan among 1655 individuals from the general population found that 64.8% of the participants stigmatize infected people and those exposed to infected people [35]. The COVID-19 crisis has created pervasive feelings of negativity and stigmatization in society and has led people to avoid getting in contact or connecting with others [60]. In certain cases, this situation has led to increased prejudice and discrimination, even hostility [60]. Also, individuals with COVID-19 may mask their symptoms to prevent marginalization and stigma [32]. This reactive behavior together with the stress caused by obscuring symptoms makes it easier for infectious diseases to spread, especially among those with mild symptoms who avoid seeking medical treatment and behave as normal to hide their illness [32].

Our results revealed that the fear of COVID-19 provokes higher discrimination stigma, in agreement with those of a study among 1687 adults from the general Columbian population, showing that high fear of COVID-19 was related to high stigma [61]. Our findings are also consistent with those of previous studies, reporting an association between the levels of fear, stigma, and discrimination in past outbreaks of infectious diseases [62]. It is well known that fear and anxiety about a disease can lead to higher stigma such as negative behaviors toward others and beliefs about persons, locations, or things [63]. Throughout history, numerous contagious diseases have been stigmatized, such as Ebola and sexually transmitted diseases like HIV/AIDS. Over the past few years, severe acute respiratory syndrome (SARS) has emerged as a new feared disease, creating substantial stigmatization [64–66], as is the case with COVID-19, where the fear of the unknown and insufficient knowledge about the virus and its lethality stigmatize the illness [18, 67]. Increased fear adds to the risk of stigma, hence the importance of raising awareness about COVID-19. Warning about negative behaviors and giving hope by talking about people who recovered from COVID-19, supporting stigmatized groups, and spreading good news will help fight the stigma and increase empathy towards COVID-19 patients while decreasing fear and stigma [68].

In our study, a higher level of knowledge was associated with lower stigma. The same result was found in Chinese research, where participants with adequate knowledge reported lower levels of stigma toward COVID-19 patients, and those who easily found and understood information about COVID-19 expressed lower stigma [69]. An Egyptian study also demonstrated that a higher knowledge score was significantly correlated with lower stigma discrimination scores [34]. Stigma increases with insufficient knowledge about how COVID-19 is transmitted, treated, and prevented [70]. In the general population, it is correlated with inadequate awareness and inconsistent facts regarding the transmission of COVID-19 [70]. Indeed, people with better knowledge have more information about emerging infectious diseases, are less anxious, and less likely to stigmatize [67]. Non-discriminatory behaviors are associated with enhanced COVID-19-related awareness, beliefs, and behaviors, and decreased stigma [71].

Regarding religiosity, our results showed that Druze and Muslims had a higher stigma than Christians. In Lebanon, the main religions are Islam (61% of the population) followed by Christianity (33.7%) [72]. The Muslim faith is a collectivist community with unique values and beliefs, depending on individual experience and group view; these beliefs can delay or encourage stigma [73]. Also, the exaggerated negative reactions of society against the Muslims and the Druze could be attributed to the role of media. Inaccurate reports spread negative perceptions about these two communities [74]. A similar study in India has shown that the entire Muslim community was stigmatized as the spreader of the virus, using expressions such as “Corona terrorism,” “enemies of humanity” and “Corona Jihad” [75]. In Turkey and the United Kingdom, conspiracy theories circulating on social media blamed Muslim immigrants for the virus [76, 77]. The political and religious issues in Lebanon contribute in favor of the stigma based on religious identity [78]. As the first declared case of COVID-19 in the country was coming from Iran, the area and the community associated with the patient were stigmatized [78]. With new cases diagnosed, the stigma was redirected to other patients of other faiths and residential areas [5].

Our findings revealed that fear and anxiety toward COVID-19, being diagnosed with COVID-19, and having a family member with COVID-19 mediated the association between knowledge and discrimination stigma, with the absence of a similar framework, exploring the relationships between these factors, in the literature. Most health-related stigma frameworks investigate psychological pathways, focusing either on persons experiencing stigma, those perpetrating it, or both [79–83]. A study among 1500 participants from the general Korean population has used a structural model to evaluate the effects of mass media usage and the level of knowledge on anxiety, and the mediating effect of fear of infection and prejudice against infected people [84]. It found that COVID-19 knowledge has a major preventive effect on fear of infection, discrimination toward infected persons, and anxiety [84]. Misconceptions about the disease and actions based on these misconceptions have been reported to contribute to a negative perception of infected people and lead to stigma or prejudice against them. Lack of awareness and knowledge about COVID-19 increases anxiety and fear by inducing negative emotions such as fear of infected persons [85–87]. Studies have found a correlation between the level of knowledge about infectious diseases and discriminatory thoughts against infected persons [88–90]. Also, it is well established that having a friend or family member who has tested positive is a factor that positively affects stigma [91]. Indeed, having a family member with COVID-19 has been related to lower levels of perceived risk and anxiety [92] and reduced need for social distance, as well as lower discrimination behaviors [66, 93]. Those already diagnosed with COVID-19 will acquire sufficient knowledge and a better understanding of the disease, which could reduce the level of stigma and discriminatory behaviors [66]. Also, patients with COVID-19 and their families may have experienced stigma, feeling judged by others, and marginalized from the community [94]. These feelings and behaviors could mitigate the stigma toward the disease. Having adequate knowledge about COVID-19 can reduce stigma towards infected people and ultimately reduce anxiety. Further studies are needed to identify variables that promote and mediate the stigmatization process of individuals. Psychosocial distress such as depression, stress, anger and anxiety expressed among COVID-19 patients and survivors, must be addressed in future studies. In addition to the assessment of personality traits and self-esteem that could affect the level of stigma. Social support is critical in reducing the harmful impacts of stigma, which can lead to illness transmission and social instability.

### Limitations

This study has several limitations. First, its cross-sectional design cannot verify the causal relationship between stigma-related variables. Second, the study relies on self-reporting data where the answers of participants about their stigmatic attitudes may be biased because of social desirability. Third, due to social distancing measures during our investigation, we used a snowball approach rather than a representative method for sampling. Also, the instruments used to measure stigma were specifically designed for this study, in the absence of a specific tool to assess COVID-19-related stigma. Also, personality traits were not taken into account. Residual confounding bias is also possible since there might be stigma-related factors that were not assessed in this study.

## Conclusion

Our main findings indicate that a considerable proportion of the Lebanese population have stigma discrimination behaviors toward COVID-19 patients and that those who had the virus experienced COVID-19-related stigmatization. Knowledge was associated with lower stigma while the fear of COVID-19 was related to higher stigma. Thus, public health education and raising community and media awareness about COVID-19 are necessary to reduce stigma. Providing targeted psychological support to citizens during a pandemic is also warranted.

## Supplementary Information

**Additional file 1. Supplementary file 1**. Stigma and COVID-19 questionnaire.** Supplementary file 2: Table 1**. Promax rotated matrix of stigma discrimination scale.** Supplementary file 2: Table 2**. Promax rotated matrix of self-stigma.** Supplementary file 3: Table 1**. Johnson-Neyman analysis plots.** Supplementary file 3: Table 2**. Plots of simple slope.** Supplementary file 3: Figure 1**. Johnson-Neyman model 1.** Supplementary file 3: Figure 2**. Plot of simple slopes model 1.** Supplementary file 3: Figure 3**. Johnson-Neyman model 2.** Supplementary file 3: Figure 4**. Plot of simple slopes model 2.** Supplementary file 3: Figure 5**. Johnson-Neyman model 3.** Supplementary file 3: Figure 6**. Plot of simple slopes model 3.** Supplementary file 3: Figure 7**. Johnson-Neyman model 4.** Supplementary file 3: Figure 8**. Plot of simple slopes model 4.

## Data Availability

Data can be made available under reasonable request form the corresponding author.

## Declarations

### Accordance statement

All methods were carried out in accordance with relevant guidelines and regulations.
