# Supplementary material for: Stigma toward people with COVID-19 among the Lebanese population: a cross-sectional study of correlates and mediating effects
Source: BMC Psychol. 2021 Oct 22;9:164. doi: 10.1186/s40359-021-00646-y (PMC8532089; doi:10.1186/s40359-021-00646-y)
Supplement: Supplementary file 1 — Additional file 1. Supplementary file 1. Stigma and COVID-19 questionnaire. Supplementary file 2: Table 1. Promax rotated matrix of stigma discrimination scale. Supplementary file 2: Table 2. Promax rotated matrix of self-stigma. Supplementary file 3: Table 1. Johnson-Neyman analysis plots. Supplementary file 3: Table 2. Plots of simple slope. Supplementary file 3: Figure 1. Johnson-Neyman model 1. Supplementary file 3: Figure 2. Plot of simple slopes model 1. Supplementary file 3: Figure 3. Johnson-Neyman model 2. Supplementary file 3: Figure 4. Plot of simple slopes model 2. Supplementary file 3: Figure 5. Johnson-Neyman model 3. Supplementary file 3: Figure 6. Plot of simple slopes model 3. Supplementary file 3: Figure 7. Johnson-Neyman model 4. Supplementary file 3: Figure 8. Plot of simple slopes model 4. [file 40359_2021_646_MOESM1_ESM.docx]

**Supplementary file:**

**Stigma Toward People with COVID-19 Among the Lebanese Population: A Cross-Sectional Study of Correlates and Mediating Effects**

**Chadia Haddad^1,2,3^, Sandrella Bou Malhab^3^, Diana Malaeb^3,4^, Hala Sacre^3^, Danielle Saadeh^3,5^, Vanessa Mourtada^6^, Pascale Salameh^3,7,8^**

1. Research department, Psychiatric Hospital of the Cross, Jal Eddib, Lebanon
2. INSERM, Univ. Limoges, IRD, U1094 Tropical Neuroepidemiology, Institute of Epidemiology and Tropical Neurology, GEIST, Limoges, France
3. INSPECT-LB (Institut National de Santé Publique, d’Épidémiologie Clinique et de Toxicologie-Liban), Beirut, Lebanon
4. School of Pharmacy, Lebanese International University, Beirut, Lebanon
5. Faculty of Public Health, Lebanese University, Beirut, Lebanon
6. Faculty of Letters and Human Sciences, Lebanese University, Fanar, Lebanon
7. Faculty of Pharmacy, Lebanese University, Beirut, Lebanon
8. University of Nicosia Medical School, Nicosia, Cyprus

**Corresponding author:** Chadia Haddad. Psychiatric Hospital of the Cross, P.O. Box 60096, Jall-Eddib, Lebanon. Email address: Chadia_9@hotmail.com.

**Supplementary file 1:**

**Stigma and COVID-19 questionnaire**

**As the COVID-19 pandemic progresses worldwide, the fear of infection increases and, with it, the stigma-discrimination of people, adding to the problem. This study is conducted by a group of academic researchers and focuses on the societal perception toward COVID-19. All the information gathered is anonymous and will be treated confidentially. Your participation in this study is voluntary.**

**Completing the questionnaire requires 10 to 15 minutes and indicates your consent to participate.**

**Thank you in advance for your time and participation.**

**SECTION 1: DEMOGRAPHIC AND OTHER CHARACTERISTICS OF THE SAMPLE**

1. Age_________________
2. Gender

Male

Female

1. Marital status

Married

Single

Widow

Divorced

1. Education level

Illiterate

Primary

Complementary

Secondary

University studies

1. Monthly income

No income

Low (< 1.500.000 LL)

Intermediate (1.500.000-3.000.000 LL)

High (>3.000.000 LL)

1. Employment status
   - - Medical field (frontline contact with COVID-19 patients)
     - Medical field (non- frontline contact)
     - Non-medical
     - Unemployed
2. Do you have a family member who works in the medical field (first line contact with COVID-19 patients)

Yes

No

1. Living place

Rural

Urban

1. Area of residence

Mount Lebanon

Beirut

North

South

Beqaa

Akkar

Nabatieh

Baalbak / hermel

1. Religion

Christian

Muslim

Druze

Atheist

Other______

Refuse to answer

1. What is the number of persons living in the house, including you: --------------?
2. What is the number of rooms in your house, excluding kitchen and bathrooms: ----------------?
3. Have you been diagnosed with COVID-19?

Yes

No

I do not know

1. Have you been tested for COVID-19?

Yes

No

1. Do you have a family member who works in the medical field (first line contact with COVID-19 patients)

Yes

No

I do not know

1. Have you been quarantined (restriction on the movement) ?

Yes

No

1. Have you had indirect contact (being in the same place, contact with the surfaces or objects used by the infected person) with someone diagnosed with COVID-19?

Yes

No

I do not know

1. Have you had direct/close contact (less than 2 meters for 15 minutes) with someone who have COVID-19?

Yes

No

I do not know

1. Have you had direct/close contact with someone suspected of having COVID-19 (having respiratory symptoms)?

Yes

No

I do not know

1. How much time (in hours) do you spend on average on COVID-19 information per day? (Including reading outbreak information on mobile or TV news, discussing the progress of the outbreak with family and friends, etc.)

- No time spent (zero time)
- Less than 30 min
- 30 min - 1 Hour
- 1 - 3 hours
- More than 3 hours

**SECTION 2: THE FOLLOWING QUESTIONS ARE RELATED TO THE IDEAS AND ATTITUDES YOU HAVE REGARDING THE COVID-19 PATIENTS OR SUSPECTED CASES.**

| **Stigma discrimination scale: Please answer the following questions** | | | | | | |
| --- | --- | --- | --- | --- | --- | --- |
|  |  | Strongly agree | Agree | Neutral | Disagree | Strongly disagree |
|  | You feel it is not worthwhile for you to serve persons who contracted COVID-19 |  |  |  |  |  |
|  | When a person with COVID-19 asks you for help, you would take the initiative to care for him/her. |  |  |  |  |  |
|  | You feel you have the right to refuse to serve people with COVID-19 in order to protect yourself from being infected. |  |  |  |  |  |
|  | People with current COVID-19 are dangerous to the society. |  |  |  |  |  |
|  | People with current COVID-19 are no different from anybody else. |  |  |  |  |  |
|  | Nobody deserves to be COVID-19 positive. |  |  |  |  |  |
|  | People with current COVID-19 should be ashamed of themselves. |  |  |  |  |  |
|  | People with current COVID-19 have nothing to feel guilty about. |  |  |  |  |  |
|  | Families of people living with COVID-19 should be ashamed. |  |  |  |  |  |
|  | People with current COVID-19 should not have the same freedoms as other people. |  |  |  |  |  |
|  | Do you relate any locations or ethnicity to the disease such as “Wuhan Virus”, “Chinese Virus” or “Asian Virus”? |  |  |  |  |  |

| **Bullying scale: Please answer the following questions** | | | | | |
| --- | --- | --- | --- | --- | --- |
|  | **Never** | **Once or twice** | **A few times (between 3 and 5)** | **Several times (between 6 and 10)** | **Many times (more than 10)** |
| 1. When someone in my surrounding have flu-like symptoms I label him as having COVID-19 |  |  |  |  |  |
| 1. I teased someone in my surrounding when I knew he tested COVID-19 positive |  |  |  |  |  |
| 1. I have called someone having COVID-19 with hurtful names |  |  |  |  |  |
| 1. I hurt someone with COVID-19/ or suspected having COVID-19 by trying to break up a friendship |  |  |  |  |  |
| 1. I have ignored someone having COVID-19 |  |  |  |  |  |
| 1. I have refused to talk with someone having COVID-19 |  |  |  |  |  |
| 1. I would not let someone having COVID-19 to join my friendship group |  |  |  |  |  |
| 1. I have told lies and/or spread rumors about someone with COVID-19 to make their friends or others not talk to him |  |  |  |  |  |
| 1. I have insulted or ridiculed someone with COVID-19 on social networks or groups like WhatsApp and Facebook to really annoy him |  |  |  |  |  |
| 1. I have sent or posted mean or hurtful pictures/videos on social networks to someone with COVID-19 |  |  |  |  |  |
| 1. I have ignored someone with COVID-19 and did not answer messages or things he shared in groups or social networks, just to make him feel bad |  |  |  |  |  |
| 1. I have eliminated or blocked someone with COVID-19 from groups to leave him/her without any friends |  |  |  |  |  |

**SECTION 3: REACTION AND FEELINGS TOWARD COVID-19**

| **Fear of COVID-19 scale: Please respond to each item and choose the best response that reflects how you feel, think, or act toward COVID-19.**  **يرجى الاجابة على كل سؤال واختيار أفضل اجابة تعكس شعورك أو تفكيرك أو تصرفك تجاه فيروس كورونا COVID-19** | | | | | | |
| --- | --- | --- | --- | --- | --- | --- |
|  | | Strongly disagree | Disagree | Neither agree nor disagree | Agree | Strongly agree |
|  | I am most afraid of coronavirus-19. |  |  |  |  |  |
|  | It makes me uncomfortable to think about coronavirus-19. |  |  |  |  |  |
|  | My hands become clammy when I think about coronavirus-19. |  |  |  |  |  |
|  | I am afraid of losing my life because of coronavirus-19. |  |  |  |  |  |
|  | When watching news and stories about coronavirus-19 on social media, I become nervous or anxious. |  |  |  |  |  |
| 6 | I cannot sleep because I’m worrying about getting coronavirus-19. |  |  |  |  |  |
| 7 | My heart races or palpitates when I think about getting coronavirus-19. |  |  |  |  |  |

| **Anxiety scale: How often have you experienced the following feelings over the last 2 weeks?**  **كم مرة عانيت من المشاعر التالية خلال الأسبوعين الماضيين؟** | | | | | | |
| --- | --- | --- | --- | --- | --- | --- |
|  | | Not at all (0) | Rare, less than a day or two (1) | Several days (2) | More than 7 days (3) | Nearly every day  over the last 2 weeks (4) |
| 1 | I felt dizzy, lightheaded, or faint, when I read or listened to news about the coronavirus |  |  |  |  |  |
| 2 | I had trouble falling or staying asleep because I was thinking about the coronavirus |  |  |  |  |  |
| 3 | I felt paralyzed or frozen when I thought about or was exposed to information about the coronavirus |  |  |  |  |  |
| 4 | I lost interest in eating when I thought about or was exposed to information about the coronavirus |  |  |  |  |  |
| 5 | I felt nauseous or had stomach problems when I thought about or was exposed to information about the coronavirus |  |  |  |  |  |

**SECTION 4: KNOWLEDGE, ATTITUDE AND PRACTICE TOWARD COVID-19**

**KNOWLEDGE SECTION**

1. **Which of the following is true about COVID-19? (check all that applies)**

- Person to person transmission can occur by droplets
- Transmission can be airborne
- Most common signs and symptoms include fever, diarrhea, and dyspnea
- I do not know

1. **For how long should a person be isolated in case of COVID-19 infection suspicion (mild symptoms or contact with an infected persons)?**

- 7 days
- 10 days
- 14 days
- 20 days
- >20 days
- I do not know

1. **Can someone who has been quarantined for COVID-19 spread the illness to others?**

- No, if the quarantine period is less than 14 days
- No, if the quarantine period is 14 days or more
- I do not know

1. **What are the steps to take to protect yourself? (check all that applies)**

- Wash your hands with soap and water for at least 10 seconds
- Wash your hands with soap and water for at least 20 seconds
- Avoid close contact; put distance between yourself and other people (1.5-2 meters)
- Wear a facemask and stay home if you have any respiratory symptom
- No need to clean and disinfect solid objects (tables, doorknobs, desks, phones, etc.)
- I do not know

1. **Can a person test negative and later test positive for COVID-19?**

- Yes
- No
- I do not know

1. **If a suspected person tests negative but has no symptoms (check all that applies):**

- It is definitely a true negative
- It can be a false negative in the pre-symptomatic phase
- I do not know how to interpret this test result, I refer to a specialist

1. **Is the person at risk if he/she goes to a funeral of someone who died of COVID-19?**

- Yes, since he will meet the dead person close contacts
- No known risk currently
- I do not know

1. **When can Confirmed COVID-19 cases be released from quarantine?**

- Following one negative PCR test after resolution of symptoms
- Following two negative PCR tests 24 hours apart after resolution of symptoms
- Following four negative PCRs on three consecutive days after resolution of symptoms
- I do not know

1. **Do you think you should avoid contact with pets or other animals if you are sick with COVID-19?**

- Yes
- No
- I do not know

1. **Who are the people most vulnerable to COVID-19? (Check all that applies)**

- Elderly
- People with underlying illness and co-morbidities
- Children
- Adolescents
- Adults

1. **What are the most common symptoms related to COVID-19?**

- Fever, productive cough, rhinorrhea
- Fever, dry cough, dyspnea
- Fever, diarrhea, pharyngitis
- None of the above
- I do not know

1. **Which of the diseases below are due to coronavirus? (Check all that applies)**

- Middle East respiratory syndrome (MERS)
- Severe acute respiratory syndrome coronavirus 2 (SARS-CoV-2)
- Influenza A (H1N1)
- Severe acute respiratory syndrome (SARS)
- I don’t know

1. **Is coronavirus the same as the common flu?**

- Yes
- No
- I don't know

1. **What is the incubation period of COVID-19?**

- 1 - 14 days
- 1 - 3 months
- 2 - 21 days
- I don't know

1. **Mode of transmission of coronavirus**

- Air droplets (from patient sneezing/coughing)
- Close contact with people who have the virus
- Contact with contaminated surfaces
- Mosquitos/flies bites
- I don't know

1. **Is hand washing important?**

- Yes
- No
- Maybe
- I don't know

1. **For how long should you wash your hands**

- 5minutes
- 1 minute to 3 minutes
- 20 seconds to 1 minute
- 3 minutes to 5 minutes
- Less than 20 seconds
- I don't know

1. **Can a person infected with coronavirus get infected a second time?**

- Yes
- No
- Maybe

1. **Can a person recovered from COVID-19 transmit the infection to others?**

- Yes
- No
- Maybe
- I don't know

1. **Can you catch the virus from surfaces and tools contaminated with COVID-19**

Maybe

No

I do not know

| PRACTICE SECTION | | | | | |
| --- | --- | --- | --- | --- | --- |
|  | Never | Rarely | Neutral | Occasionally | Always |
| 1. In the last few days, have you worn a mask when you were in a crowded place? |  |  |  |  |  |
| 1. In the last few days, have you implemented physical distancing when you were in the crowd? |  |  |  |  |  |
| 1. In the last few days, have you used hand sanitizer when you were in crowded places? |  |  |  |  |  |
| 1. In the last few days, have you washed your hands with soap after going to a crowded place? |  |  |  |  |  |
| 1. Do you use tissues or cover your mouth during coughing/sneezing? |  |  |  |  |  |
| 1. Do you replace the face mask after a single use |  |  |  |  |  |
| 1. Do you avoid touching face and eyes? |  |  |  |  |  |
| 1. Do you maintain social distancing (or home quarantine)? |  |  |  |  |  |
| 1. Do you eat healthy food focusing on outbreak? |  |  |  |  |  |
| 1. Do you obey all government rules related to the COVID? |  |  |  |  |  |
| 1. Do you Clean/disinfect your mobile phone |  |  |  |  |  |
| 1. Do you avoid contact with people at risk |  |  |  |  |  |
| 1. Do you avoid groups |  |  |  |  |  |
| 1. Do you avoid shaking hands |  |  |  |  |  |
| 1. Do you stay at home when it is requested by the government |  |  |  |  |  |
| 1. Do you stay at home with symptoms |  |  |  |  |  |
| 1. If a friend or relative of yours were to have frequent contact with people with COVID-19, you prefer not to meet him/her. |  |  |  |  |  |
| 1. Even if you only had to speak to a person with COVID-19, you would wear a mask to prevent infection. |  |  |  |  |  |
| 1. For reasons of general safety, you think you should not get near a person with COVID-19. |  |  |  |  |  |
| 1. You are not against serving persons with COVID-19, but would try your best not to get too close to them. |  |  |  |  |  |
| 1. People with current COVID-19 should be isolated and cannot actively participate in the social events in this community. |  |  |  |  |  |
| 1. A person with COVID-19 should not be allowed to work with other people. |  |  |  |  |  |
| 1. Families of people living with COVID-19 should be isolated. |  |  |  |  |  |
| 1. It is reasonable for an employer to exclude/isolate a person with COVID-19. |  |  |  |  |  |

| ATTITUDES SECTION | | | |
| --- | --- | --- | --- |
|  | Disagree | Not sure | Agree |
| 1. Do you think social distancing/self-isolation is an effective measure to reduce the spread of COVID-19? |  |  |  |
| 1. Do you think that regular hand washing, maintaining social distancing and use of masks can protect you from coronavirus? |  |  |  |
| 1. Do you think lockdown will be helpful in controlling the coronavirus disease |  |  |  |
| 1. Keeping up with the information regarding the government’s call for COVID-19 preventive efforts is important for the community |  |  |  |
| 1. People with COVID-19 should not be stigmatized in society |  |  |  |
| 1. People with COVID-19 who isolate themselves show that they have a responsibility in preventing the transmission of COVID-19 |  |  |  |

**Section 5: If you have been diagnosed (PCR positive) or suspected of having COVID-19 (flu-like symptoms or contact with positive cases), please fill out the following questions**

| **Self-Stigma Scale For People with COVID-19** | | | | | | |
| --- | --- | --- | --- | --- | --- | --- |
|  | | Strongly Disagree | Disagree | Neutral | Agree | Strongly Agree |
|  | I feel guilty because of being isolated |  |  |  |  |  |
|  | I feel blamed by relatives or friends |  |  |  |  |  |
|  | I feel ashamed of being isolated |  |  |  |  |  |
|  | I try to hide being quarantined |  |  |  |  |  |
|  | I try to avoid going out |  |  |  |  |  |
|  | I do not disclose to anyone about the feeling |  |  |  |  |  |
|  | People talk behind my back |  |  |  |  |  |
|  | People avoid touching me and direct contact with me |  |  |  |  |  |
|  | People feel uncomfortable when around |  |  |  |  |  |
|  | People have physically backed away from  me when they learned I have COVID-19 |  |  |  |  |  |
|  | People seemed afraid of me once they learned I have COVID-19 |  |  |  |  |  |
|  | I have been hurt by how people reacted to  learning I have COVID-19 |  |  |  |  |  |
|  | Since learning I have COVID-19, I worry about people discriminating against me |  |  |  |  |  |
|  | Some people I care about stopped calling after learning that I have COVID-19 |  |  |  |  |  |
|  | Some people who knew that I have COVID-19 have grown more distant |  |  |  |  |  |

**Supplementary file 2:**

| **Supplementary table 1: Promax rotated matrix of stigma discrimination scale** | | | | | | |
| --- | --- | --- | --- | --- | --- | --- |
| **Factor** | **Item** | **Factor 1** | **Factor 2** | **Factor 3** | **Factor 4** | **H2 communalities** |
| **Families of people living with COVID-19 should be ashamed** | 18 | 0.877 |  |  |  | 0.795 |
| **People with COVID-19 should be ashamed of themselves** | 13 | 0.873 |  |  |  | 0.775 |
| **Do you relate any locations or ethnicity to the disease such as “Wuhan Virus”, “Chinese Virus” or “Asian Virus”?** | 26 | 0.633 |  |  |  | 0.446 |
| **You feel it is not worthwhile for you to serve persons who contracted COVID-19** | 1 | 0.432 |  |  |  | 0.456 |
| **People with COVID-19 are dangerous to the society** | 9 |  | 0.810 |  |  | 0.677 |
| **People with COVID-19 should not have the same freedoms as other people** | 22 |  | 0.680 |  |  | 0.495 |
| **People with COVID-19 are no different from anybody else** | 11 |  | 0.659 |  |  | 0.642 |
| **You feel you have the right to refuse to serve people with COVID-19 in order to protect yourself from being infected** | 4 |  |  | 0.804 |  | 0.679 |
| **When a person with COVID-19 asks you for help, you would take the initiative to care for him/her.** | 2 |  |  | 0.770 |  | 0.658 |
| **Nobody deserves to be COVID-19 positive** | 12 |  |  |  | 0.736 | 0.586 |
| **People with COVID-19 have nothing to feel guilty about** | 14 |  |  |  | 0.708 | 0.516 |
| Percentage variance explained | | 22.88% | 15.58% | 12.46% | 10.20% |  |
| Chronbach alpha= 0.565 | | | | | | |
| Kaiser-Meyer-Olkin (KMO)= 0.641 | | | | | | |
| Bartlett’s test of sphericity p<0.001 | | | | | | |

| **Supplementary table 2: Promax rotated matrix of self-stigma** | | | | | |
| --- | --- | --- | --- | --- | --- |
| **Factor** | **Item** | **Factor 1** | **Factor 2** | **Factor 3** | **H2 communalities** |
| **People avoid touching me and direct contact with me** | 8 | 0.965 |  |  | 0.859 |
| **People have physically backed away from me when they learned I have COVID-19** | 10 | 0.903 |  |  | 0.868 |
| **I try to avoid going out** | 5 | 0.883 |  |  | 0.682 |
| **People seemed afraid of me once they learned I have COVID-19** | 11 | 0.823 |  |  | 0.825 |
| **People feel uncomfortable when around** | 9 | 0.805 |  |  | 0.784 |
| **People talk behind my back** | 7 | 0.672 |  |  | 0.608 |
| **Some people who knew that I have COVID-19 have grown more distant** | 15 |  | 0.863 |  | 0.742 |
| **Since learning I have COVID-19, I worry about people discriminating against me** | 13 |  | 0.862 |  | 0.685 |
| **Some people I care about stopped calling after learning that I have COVID-19** | 14 |  | 0.857 |  | 0.775 |
| **I have been hurt by how people reacted to learning I have COVID-19** | 12 |  | 0.634 |  | 0.691 |
| **I feel blamed by relatives or friends** | 2 |  | 0.494 |  | 0.682 |
| **I try to hide being quarantined** | 4 |  |  | 0.928 | 0.825 |
| **I feel ashamed of being isolated** | 3 |  |  | 0.882 | 0.790 |
| **I do not disclose to anyone about the feeling** | 6 |  |  | 0.757 | 0.618 |
| **I feel guilty because of being isolated** | 1 |  |  | 0.615 | 0.587 |
| Percentage variance explained | | 46.685% | 16.541% | 10.250% |  |
| Chronbach alpha= 0.917 | | | | | |
| Kaiser-Meyer-Olkin (KMO) =0.717 | | | | | |
| Bartlett’s test of sphericity p<0.001 | | | | | |

**Supplementary file 3**

**Moderation analysis results**

| **Table 1** Johnson-Neyman analysis plots | | |
| --- | --- | --- |
| **Model 1: Taking the stigma discrimination scale as the dependent variable and the fear from COVID-19 scale as a mediation factor.** | | |
| Variable | Regression coefficient | Region of significance |
| Stigma discrimination (Y) |  | Simple Slope crosses over the significance barrier at m1=5.63 and m2=18.07 |
| Intercept (γ_0_) | 33.64 |  |
| Knowledge (X) (γ_1_) | -0.48 |  |
| Fear from COVID-19 (M) (γ_2_) | -0.23 |  |
| Interaction X*M (γ_3_) | 0.02 |  |
| M |  |  |
| Mean (SD) | 17.48 (5.54) |  |
| **Model 2: Taking the stigma discrimination scale as the dependent variable and the anxiety from COVID-19 scale as a mediation factor.** | | |
| Variable | Regression coefficient | Region of significance |
| Stigma discrimination (Y) |  | Simple Slope crosses over the significance barrier at m1=1.64 and m2=8.62 |
| Intercept (γ_0_) | 27.29 |  |
| Knowledge (X) (γ_1_) | -0.06 |  |
| Anxiety from COVID-19 (M) (γ_2_) | 1.33 |  |
| Interaction X*M (γ_3_) | 0.06 |  |
| M |  |  |
| Mean (SD) | 1.17 (2.45) |  |
| **Model 3: Taking the stigma discrimination scale as the dependent variable and being diagnosed with COVID-19 as a mediation factor.** | | |
| Variable | Regression coefficient | Region of significance |
| Stigma discrimination (Y) |  | Simple Slope crosses over the significance barrier at m1=0.08 and m2=0.48 |
| Intercept (γ_0_) | 29.21 |  |
| Knowledge (X) (γ_1_) | -0.13 |  |
| Diagnosis with COVID-19 (M) (γ_2_) | 4.41 |  |
| Interaction X*M (γ_3_) | -0.37 |  |
| M |  |  |
| Mean (SD) | 0.10 (0.30) |  |
| **Model 4: Taking the stigma discrimination scale as the dependent variable and family history of COVID-19 as a mediation factor.** | | |
| Variable | Regression coefficient | Region of significance |
| Stigma discrimination (Y) |  | Simple Slope crosses over the significance barrier at m1=0.10 and m2=0.70 |
| Intercept (γ_0_) | 30.05 |  |
| Knowledge (X) (γ_1_) | -0.15 |  |
| Family history of COVID-19 (M) (γ_2_) | 0.22 |  |
| Interaction X*M (γ_3_) | -0.11 |  |
| M |  |  |
| Mean (SD) | 0.35 (0.55) |  |

**Table 2** Plots of simple slope

| **Model 1** | | | | |
| --- | --- | --- | --- | --- |
|  | **Simple slope (SE)** | **95% CI** |  |  |
| **Low fear (M)** | -0.27 (0.12) | -0.50; -0.03 |  |  |
| **High fear (M)** | -0.07 (0.10) | -0.27; 0.14 |  |  |
|  | **X Value** | **X Labels** | **Low M (-1 SD)** | **High M (+1 SD)** |
| **Low knowledge (X)** | 16.88 | **Low X (-1 SD)** | 26.42 | 27.74 |
| **High knowledge (X)** | 23.90 | **High X (+1 SD)** | 24.54 | 26.76 |
| **Model 2** | | | | |
|  | **Simple slope (SE)** | **95% CI** |  |  |
| **Low anxiety (M)** | 0.009 (0.12) | -0.22; 0.24 |  |  |
| **High anxiety (M)** | -0.27 (0.12) | -0.50;-0.04 |  |  |
|  | **X Value** | **X Labels** | **Low M (-1 SD)** | **High M (+1 SD)** |
| **Low knowledge (X)** | 16.88 | **Low X (-1 SD)** | 27.52 | 26.63 |
| **High knowledge (X)** | 23.90 | **High X (+1 SD)** | 25.63 | 25.71 |
| **Model 3** | | | | |
|  | **Simple slope (SE)** | **95% CI** |  |  |
| **Negative COVID-19 (Low M)** | -0.06 (0.12) | -0.29; 0.16 |  |  |
| **Positive COVID-19 (High M)** | -0.28 (0.14) | -0.54;-0.01 |  |  |
|  | **X Value** | **X Labels** | **Low M (-1 SD)** | **High M (+1 SD)** |
| **Low knowledge (X)** | 16.88 | **Low X (-1 SD)** | 27.37 | 26.30 |
| **High knowledge (X)** | 23.90 | **High X (+1 SD)** | 24.34 | 26.66 |
| **Model 4** | | | | |
|  | **Simple slope (SE)** | **95% CI** |  |  |
| **Negative COVID-19 family history (Low M)** | -0.13 (0.11) | -0.35; 0.09 |  |  |
| **Positive COVID-19 family history (High M)** | -0.25 (0.15) | -0.54; 0.03 |  |  |
|  | **X Value** | **X Labels** | **Low M (-1 SD)** | **High M (+1 SD)** |
| **Low knowledge (X)** | 16.88 | **Low X (-1 SD)** | 27.79 | 26.02 |
| **High knowledge (X)** | 23.90 | **High X (+1 SD)** | 26.87 | 24.25 |

**MODEL 1**

Figure 1 Johnson-Neyman model 1

Figure 2 Plot of simple slopes model 1

**MODEL 2**

Figure 3 Johnson-Neyman model 1

Figure 4 Plot of simple slopes model 1

**MODEL 3**

Figure 5 Johnson-Neyman model 1

Figure 6 Plot of simple slopes model 1

**MODEL 4**

Figure 7 Johnson-Neyman model 1

Figure 8 Plot of simple slopes model 1
